# Performance of Denitrifying Microbial Fuel Cell with Biocathode over Nitrite

**DOI:** 10.3389/fmicb.2016.00344

**Published:** 2016-03-22

**Authors:** Huimin Zhao, Jianqiang Zhao, Fenghai Li, Xiaoling Li

**Affiliations:** ^1^Department of Environmental Engineering, School of Environmental Science and Engineering, Chang'an UniversityXi'an, China; ^2^Department of Chemistry and Chemical Engineering, Heze UniversityHeze, China

**Keywords:** microbial fuel cell, autotrophic denitrification, nitrite, nitration, biocathode

## Abstract

Microbial fuel cell (MFC) with nitrite as an electron acceptor in cathode provided a new technology for nitrogen removal and electricity production simultaneously. The influences of influent nitrite concentration and external resistance on the performance of denitrifying MFC were investigated. The optimal effectiveness were obtained with the maximum total nitrogen (TN) removal rate of 54.80 ± 0.01 g m^−3^ d^−1^. It would be rather desirable for the TN removal than electricity generation at lower external resistance. Denaturing gradient gel electrophoresis suggested that *Proteobacteria* was the predominant phylum, accounting for 35.72%. *Thiobacillus* and *Afipia* might benefit to nitrite removal. The presence of nitrifying *Devosia* indicated that nitrite was oxidized to nitrate via a biochemical mechanism in the cathode. *Ignavibacterium* and *Anaerolineaceae* was found in the cathode as a heterotrophic bacterium with sodium acetate as substrate, which illustrated that sodium acetate in anode was likely permeated through proton exchange membrane to the cathode.

## Introduction

Microbial fuel cell (MFC) possesses great potential in the application of wastewater treatment because of its unique capability of converting the chemical energy of organic waste into electrical energy (Logan et al., 2006). It has been proved that both nitrate and nitrite can be removed from wastewater as electron acceptors in the cathode of MFCs through electrochemical reduction or autotrophic denitrification (Wang et al., 2009; Desloover et al., 2011; Zhao et al., 2011). In MFC, the organic substrates are oxidized by exoelectrogenic microbes in the anode chamber to produce electrons and protons. Electrons produced are transferred through the external circuit to the cathode while protons move through the proton exchange membrane to the cathode, where they combine to an electron acceptor (e.g., nitrate or nitrite) to complete the circuit (Van Doan et al., 2013). Virdis et al. (2008) discovered that nitrite could serve as an efficient terminal electron acceptor at the cathode of MFC, which further reduced the carbon-nitrogen ratio demand. The similar results were also demonstrated by Puig et al. (2011) and Desloover et al. (2011). The biotic cathode using nitrite as an electron acceptor showed a TN removal percentage of 48% and a removal rate of 7.6 g (${\text{NO}}_{2}^{-}$-N) m^−3^ d^−1^ during the 4 h continuing mode of operation (Puig et al., 2011). Although the TN removal rate via cathodic (autotrophic) denitrification in MFC is generally lower than that via heterotrophic denitrification, it is very important to notice that autotrophic microbes need few carbon source and their slow growth results in small sludge production (Wang et al., 2009; Zhao et al., 2011). Consequently, autotrophic denitrifying MFCs are promising technologies to treat low organic carbon wastewater, which greatly reduced the dependence on carbon in denitrifying process.

However, nitrite is oxidized easily by biological or electrochemical processes which significantly degraded the TN removal and electricity generation efficiency. Puig et al. (2011) found that about 52% nitrite oxidized to nitrate in the MFC cathode at an external resistance of 100 Ω. And he speculated that disappeared nitrite was oxidized by nitrite oxidizing bacteria (NOBs) or by other electrochemical processes. Li et al. (2014) also found that about 80% nitrite oxidized to nitrate. To inhibit the nitrification, one way was to add sodium azide in the cathode (Guisasola et al., 2005; Puig et al., 2011), the other way was to change the operating conditions of a cathode chamber(e.g, external resistance and HRT and temperature; Li et al., 2014). In order to further clarify these influencing factors of denitrifying MFC and the mechanism of nitrite conversion to nitrate in the cathode of MFC without the addition of chemical inhibitors, this study aimed to investigate the performance of the denitrification of MFC, which based on electricity generation and nitrite removal with different nitrite concentrations and external resistances in the denitrifying MFC at the long duration of the operation. PCR-DGGE was used to assess the cathode microbial community to speculate for possible reactions in the cathode.

## Materials and methods

### Structure of MFC and operation

**Figure 1:**
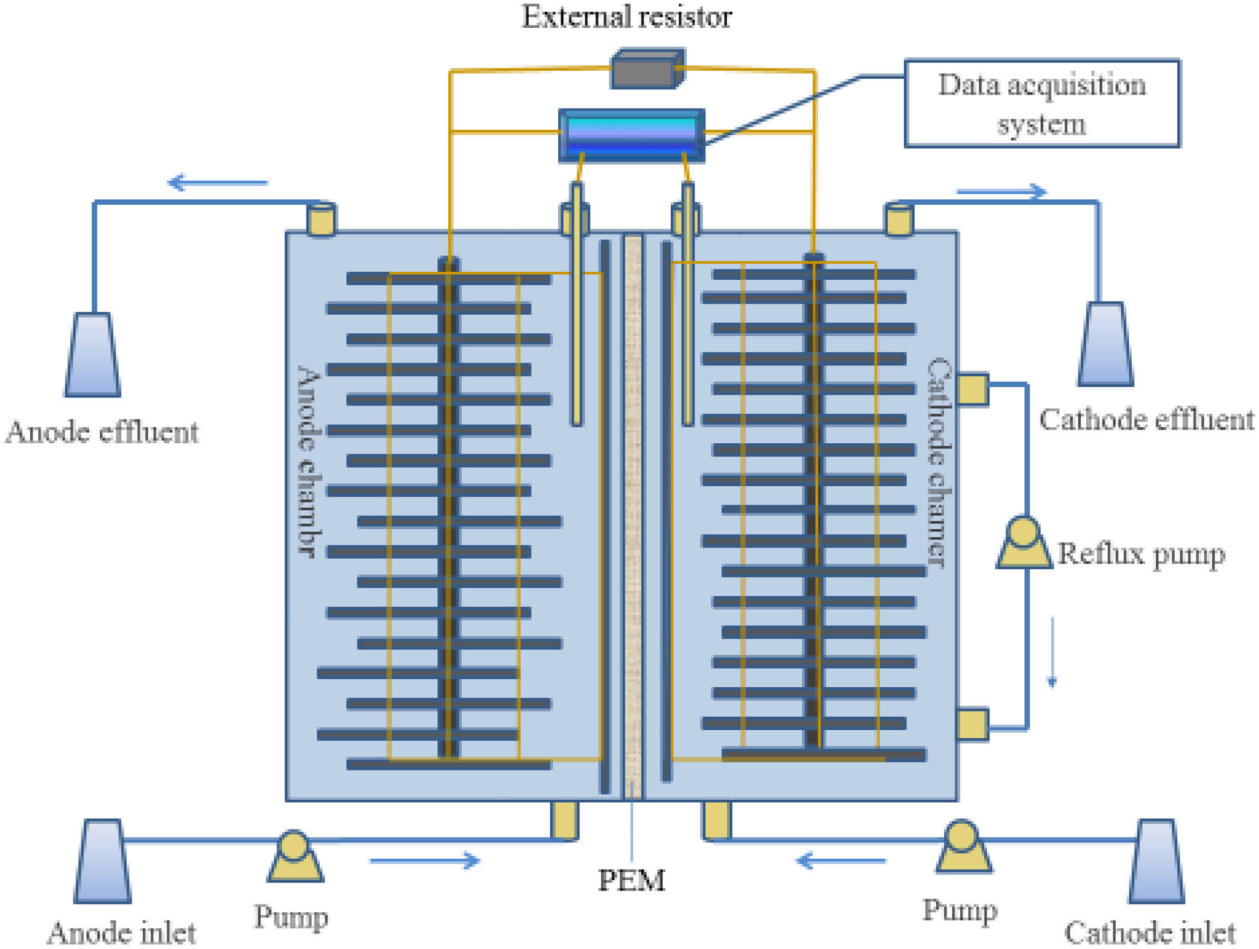
**Schematic of the double chamber MFC**.

The MFC consisting of an anode chamber and a cathode chamber placed on opposite sides of a single methacrylate rectangular chamber with dimensions of 15 cm high, 5 cm long, and 2.5 cm wide. A proton exchange membrane (nafion117, DuPont, USA) was used as a separator between anode and cathode chambers. Each chamber was filled with rectangular graphite felts (140 mm long, 11.7 mm wide, and 5 mm thick) as electrode and inserted with a graphite rod, which led to the eventual volume of 160 cm^3^ for cathodic and anodic chamber, respectively. The electrodes were sequentially washed in 1 M HCl and 1 M NaOH to remove possible metal and biomass contamination (Bond and Lovley, 2003). The cathodic and the anodic electrodes were connected to the external resistor to close the electric circuit. A Hg/Hg Cl electrode (+0.242 V vs. SHE) was used as a reference electrode placing in the cathode solution. Three peristaltic pumps (Lan Ge YZ1515X, Baoding, China) were used to continuously supply influents to anode and cathode chambers, and reflux the cathode solution. All experiments were performed at 32 ± 1°C. Figure 1 showed the schematic diagram of the MFC in this study.

The electrode material was immersed in corresponding seeding sludge (anaerobic sludge from Xi'an Hans Brewery Wastewater Treatment, China) for 48 h to absorb bacteria and then loaded in corresponding chambers. The starting procedure of the MFC was followed as reported by Virdis et al. (2008). The flow rate of influent was maintained at 3 mL h^−1^. The initial resistance was set at 1000 Ω for 15 days and then turned to 100 Ω for about a month. When the output voltage of the MFC was stable and reached 200 mV with external resistance of 100 Ω, the start-up of the MFC was considered to be successful. Then, the resistance was kept constant at 10 Ω, and maintained for 240 days. A series of experiments were performed, the performance of MFC was studied in terms of changing the nitrite nitrogen concentrations of (60, 90, and 180 mg L^−1^) at external resistance of 10 Ω. Afterward, the effect of external resistance was studied by varying external resistances in the range from 5 to 10, 25, 50, 100, and 200 Ω.

The anode solution was composed of CH_3_COONa (3.84 g L^−1^), KCl (0.13 g L^−1^), MgSO_4_·7H_2_O (0.1 g L^−1^), CaCl_2_ (0.015g L^−1^), K_2_HPO_4_·3H_2_O (8.57 g L^−1^), KH_2_PO_4_ (2.88 g L^−1^), and trace elements 1 mL L^−1^.

The cathode solution was composed of NaNO_2_ (0.15 g L^−1^), NaHCO_3_(1 g L^−1^), MgSO_4_·7H_2_O (0.1 g L^−1^), CaCl_2_ (0.015g L^−1^), K_2_HPO_4_·3H_2_O (8.57 g L^−1^), KH_2_PO_4_ (2.88 g L^−1^), and trace elements 1 mL L^−1^.

### Data calculation and analysis

The voltage (V) and cathode potentials of the MFC were monitored at 1 min intervals and 10 min averaged with a data acquisition system (Yanhua PCI1713, China). Current (I) and power (P = I·V) were determined according to Ohm's law. Power and current densities were calculated by dividing power or current by the net cathodic volume. The cathodic Coulombic efficiencies was calculated according to Logan et al. (2006). DO was determined using Hach-HQ30d (HACH, USA). The concentrations of ${\text{NO}}_{2}^{-}$-N and ${\text{NO}}_{3}^{-}$-N were measured according to standard methods (APHA, 1998). During the experimental, all analyses under the same operations were carried out more than triplicate.

### DNA analysis

After being operated stably for 9 months at the external resistance of 10 Ω and the flow rate of 3 mL h^−1^ and the nitrite nitrogen concentrations of 188 mg L^−1^, biofilm sample from the suspension liquid of the cathode was taken to be investigated with denaturing gradient gel electrophoresis (DGGE), and DNA was extracted using a fast DNA spin kit (SK8233) for soil according to the manufacturer's instructions. The bacterial 16S rRNA genes were amplified by polymerase chain reaction (PCR) techniques with the universal primers F357-GC (5′-CGC CCGCCGCGCCCCGCG CCCGGCCCGCCGCCCCGCCCCCCTACGGGAGGCAGC AG-3′) and R518 (5′-ATTACCGCG GCTGCTGG-3′). Polyacrylamide gel (8%) with a 30–60% denaturing gradient was used to separate the PCR products (7 mol L^−1^ urea and 40% formamide comprising 100% denaturant), and the PCR product was analyzed by the DGGE technology and washed with ultrapure water for flushing the gel and dye. The eight representative DGGE strips were selected by a clean scalpel to select and transfer in a 1.5 mL centrifuge tube. Then, the target DNA fragments were excised and reamplified by using the primer sets F357 (5′-CCTACGGGA GGCAGCAG-3′) and R518 (5′-ATTACC GCGGCTGCTGG-3′), and the obtained sequence was matched with the Seqmatch database for sequence alignment. The homology information of each strip was obtained by Shanghai Sangong Biological Engineering Co., Ltd. China. This process was similar to that reported by Deng et al. (2016).

## Results and discussion

### Performance of denitrification MFC with different nitrite concentrations

The results of different influent nitrite concentration at the inflow rate of 3 mL h^−1^ and the external resistance of 10 Ω and temperature of 32°C were listed in Tables 1, 2.

**Table 1:** **Electrical characteristics of the MFC with different nitrite concentrations**.

**Inflow rate (mL h^−1^)**	**Influent ${\text{NO}}_{2}^{-}$—N (mg L^−1^)**	**Cathode potential (mV)**	**Current density (A m^−3^)**	**Power density (W m^−3^)**	**Columbic efficiency (%)**
3	60.11 ± 0.34	−35.2±3.2	18.02 ± 0.81	0.518 ± 0.035	279.5 ± 32.15
3	86.65 ± 0.61	−47.22±5.3	19.25 ± 1.07	0.594 ± 0.071	231.53 ± 28.53
3	188.12 ± 2.3	−38.36±2.5	18.40 ± 0.36	0.541 ± 0.004	140.12 ± 0.71

**Table 2:** **Characteristics of denitrification with different nitrite concentrations**.

**Inflow rate (mL h^−1^)**	**Influent ${\text{NO}}_{2}^{-}$—N (mg L^−1^)**	**Effluent ${\text{NO}}_{2}^{-}$—N(mg L^−1^)**	**Δ ${\text{NO}}_{3}^{-}$—N (mg L^−1^)**	**Nitrification Percentage (%)**	**TN removal (g m^−3^ d^−1^)**	**Δ pH Effluent**
3	60.11 ± 0.34	0	0.2 ± 0.1	0.33	26.91 ± 1.72	0.85
3	86.65 ± 0.61	4.03 ± 2.17	5.03 ± 3.35	5.81	34.92 ± 2.2	1.03
3	188.12 ± 2.3	16.72 ± 0.59	49.5 ± 0.5	26.33	54.80 ± 0.01	1.18

*Δ ${\text{NO}}_{3}^{-}$-N = incremental nitrate*.

As shown in Table 1, when the flow rate was 3 mL·h^−1^, the increase of influent nitrite concentration had little benefit on current density and power density. The cathode potential decreased with the increase of current density. The cathode coulombic efficiency was higher than 100% due to other oxidizing substances (e.g., oxygen) in the cathode functioning as a terminal electron acceptor especially when the nitrite concentration of the effluent was about zero (Table 2; Xie et al., 2011). Cha et al. (2010) found that microorganism using with oxygen as electron acceptor for oxygen utilization efficiency was very high, which might compete with denitrifying microorganism and affect the cathode denitrification.

The autotrophic denitrification of nitrite to nitrogen gas in the bio-cathode can be described by the following equations (reaction 1-3; Clauwaert et al., 2007). Table 2 showed that when the flow rate was maintained at 3 mL h^−1^, with increasing concentration of the influent nitrite, the TN removal rate increased significantly from 26.91 ± 1.72 g m^−3^ d^−1^ to the maximum of 54.80 ± 0.01 g m^−3^ d^−1^; but the nitrification percentage increased from 0.33 to 26.33% meanwhile. Therefore, considering two factors of nitrification and the TN removal, we operated the MFC at the high influent nitrite of 188 mg L^−1^ which would be favorable.

(1) $$\begin{array}{l} \text{Nitrite reduction}:{\text{NO}}_{2}^{-}+e^{-}+2{\text{H}}^{+}=\text{NO}+{\text{H}}_{2}\text{O}\\ \;E^{0}=+0.350\text{V vs SHE} \end{array}$$

(2) $$\begin{array}{l} \text{Nitric oxide reduction}:\text{NO}+e^{-}+{\text{H}}^{+}=0.5{\text{N}}_{2}\text{O}+0.5\text{H}\\ \;E^{0}=+1.175\text{V vs SHE} \end{array}$$

(3) $$\begin{array}{l} \text{Nitrous oxide reduction}:\text{0}.{\text{5N}}_{2}\text{O}+e^{-}+{\text{H}}^{+}=0.5{\text{N}}_{2}\\ \;+0.5{\text{H}}_{2}\text{O}\\ \;E^{0}=+1.355\text{V vs SHE} \end{array}$$

(4) $$\text{Nitrification process}:{\text{NO}}_{2}^{-}+0.5{\text{O}}_{2}={\text{NO}}_{3}^{-}$$

Under the condition of strict measures to maintain the anoxic condition in the cathode, but the cathode still showed obvious nitrite nitrification. The small part of nitrite to nitrate transformation might be caused by biological nitrification in this study (reaction 4) because of trace oxygen in the cathode. While the other part of the nitrite transformation also might be oxidized by other electrochemical processes (Puig et al., 2011).

### Performance of denitrification MFC at different external resistances

**Figure 2:**
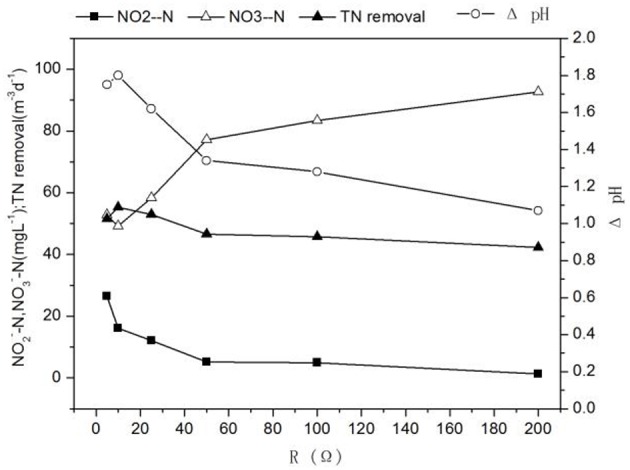
**Profiles of characteristics of cathode effluent with different external resistances**.

**Figure 3:**
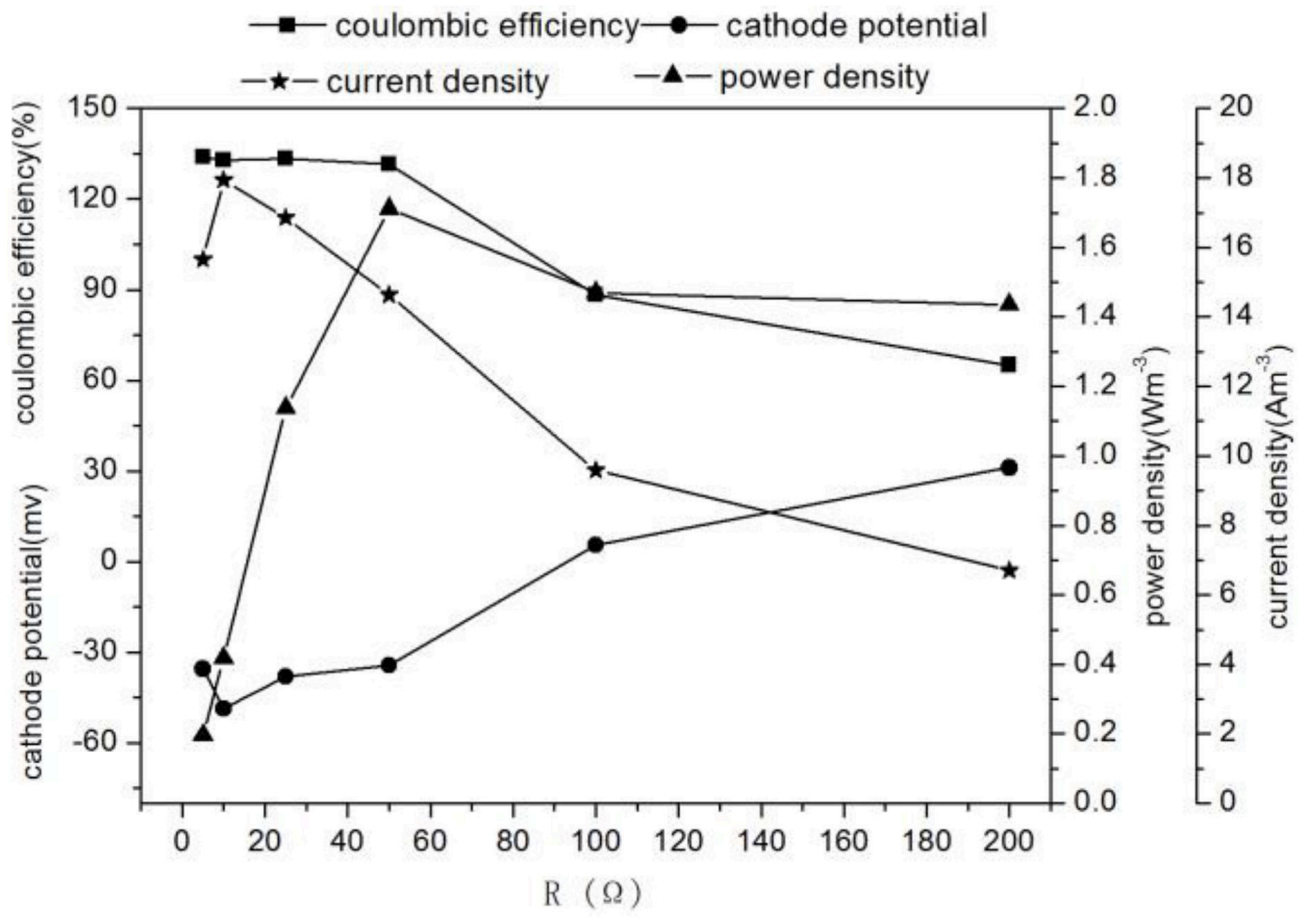
**Characteristics electricity production of MFC with different external resistances**.

When the influent nitrite nitrogen concentration and influent flow rate were 188 ± 2.3 mg L^−1^, 3 mL h^−1^, respectively. The results of denitrification at different external resistances were shown in Figures 2, 3, respectively.

Different external resistances cause different electron transfer rates and variations in microbial metabolic activities and kinetic differences in substrate utilization (Zhang et al., 2011). Usually, the pollutant removal of MFC is faster at the smaller external resistance which can reduce the extracellular electron transfer resistance and increase the electron transfer rate (Katuri et al., 2011). As external resistance was increased from 5 to 200 Ω, the concentrations of nitrite in effluent and the TN removal rate decreased significantly from 26.55 ± 0.85 to 1.26 ± 0.09 mg L^−1^ and 51.51 ± 0.17 to 42.25 ± 0.24 g m^−3^ d^−1^, whereas the nitrate concentration in effluent increased from 52.84 ± 0.48 to 92.62 ± 1.47 mg L^−1^ (Figure 2), the increase of effluent pH changed with the increase of the TN removal rate, which showed that a large external resistance was not help to denitrification. Zhang and He (2012) found that the TN removal rate increased from 51.9 to 68% with decreasing external resistance from 712 to 10 Ω in a dual chamber MFC. At the same time, the potential of cathode increased (excepting at 5 Ω) -35.49 to 31.11 mV, while the current density and the cathode coulombic efficiency decreased 15.66–6.7 Am^−3^ and 133.91 to 65.05%. The low coulombic efficiency (65.05%) indicated possible intermediate accumulation such as N_2_O and NO. Because the reduction of nitrite to N_2_ requires 3 mol electrons, whereas the reduction of nitrite to NO and N_2_O need 1 mol and 2 mol electron, respectively, which causing low current density and coulombic efficiency (Wrage et al., 2001). These results were in accordance with Virdis et al. (2010) who observed 29.2% total nitrogen conversion to N_2_O, Puig et al. (2011) also showed that the cathode coulombic efficiency was ~48%, confirming the existence of the intermediate product NxO in the process of denitrification, causing the cathode coulombic efficiency to be below 100%.

It was found that the highest power density(1.71 W m^−3^) was obtained at 50 Ω while the highest TN removal rate (54.80 ± 0.01 g m^−3^ d^−1^) was at 10 Ω (Figures 2, 3). The result implied that operation of denitrifying MFC at a lower external resistance would be desirable for the TN removal but not electricity generation (Li et al., 2013). The performance of the MFC became poor when the external resistance was turned to 5 Ω, which indicated the MFC reaching the limit current. Therefore, if the aim of the MFC was the TN removal over electricity generation for a denitrifying MFC, operation would be desirable at lower external resistance (except generating limit current).

### Identification of cathode microbial species

The microbial communities of the nitrite bio-cathodes were analyzed by DGGE. As shown in Table 3. The microbial community structure was diversity in the cathode of MFC. In addition to these bands, the bio-cathode samples contained clones that were mostly assigned to known sequences, The bacterial communities were *Devosia*(bands 6), *Pelomonas*(bands 19)*, Thiobacillus*(bands 20), and *Afipia*(bands 29) in Phylum *Proteobacteria* (35.72%), *Proteobacteria* was found to be dominative in the denitrification of MFC cathode (Karanasios et al., 2010). Kondaveeti et al. (2014) also identified several members of *Proteobacteria* and *Firmicutes* in cathodic nitrate and nitrite reduction. *Truepera*(bands 9 and 18) related to Phylum *Deinococcus–Thermus* (30.98%), *Ignavibacterium*(bands 5 and 22) similar to Phylum *Ignavibacteriae* (17.14%), and *Bellilinea*(bands 16) and *Anaerolineaceae* (bands 31) corresponding to Phylum *Chloroflexi* (16.15%).

**Table 3:** **The identifications of DGGE bands**.

**Band**	**Proportion (%)**	**Taxon**	**Similarity (%)**	**Accession**	**Phylum/Genus**
5	8.19	Uncultured bacterium	100	AY548931	*Ignavibacteriae/Ignavibacterium*
22	8.95	uncultured bacterium	93	*EU283596*	*Ignavibacteriae/Ignavibacterium*
6	4.69	*Candidatus Devosia euplotis*	81	*AJ548823*	*Proteobacteria/Devosia*
19	8.86	uncultured bacterium	88	*AB487482*	*Proteobacteria/Pelomonas*
20	8.19	uncultured bacterium	83	*FJ516975*	*Proteobacteria/Thiobacillus*
29	13.98	Afipia massiliensis	100	*AB272322*	*Proteobacteria/Afipia*
9	7.96	Uncultured bacterium	100	*EU083501*	*Deinococcus-Thermus /Truepera*
18	23.02	Uncultured bacterium	93	*FN436167*	*Deinococcus-Thermus/Truepera*
16	7.06	*Bellilinea caldifistulae*	87	*AB355078*	*Chloroflexi/Bellilinea*
31	9.09	uncultured bacterium	88	*JQ408049*	*Chloroflexi/Anaerolineaceae*

Analysis of the microbial communities newly developed on the bio-cathodes revealed that most of them have previously been demonstrated to be capable of communicating with the electrode, For example, *Afipia* and *Thiobacillus* were dominant species responsible for autotrophic denitrifying in the cathode of MFC (Kelly and Wood, 2000; La Scola et al., 2002). *Devosia* had nitrification ability contributing to the nitration phenomenon in the experiment (Vanparys et al., 2005). *Ignavibacterium* (Okamoto et al., 2013) was distinctively detected on the bio-cathode and involved in heterotrophic denitrifying bacteria. *Anaerolineaceae* was the anaerobic methanogenesis for sodium acetate as the substrate (Yamada et al., 2006). The proportion of aerobic *Truepera* and *Pelomonas* was 39.64%, which exhibited the ability of respiration with oxygen (Albuquerque et al., 2005; Chandra et al., 2011).

### Mechanism of the cathode chamber

**Figure 4:**
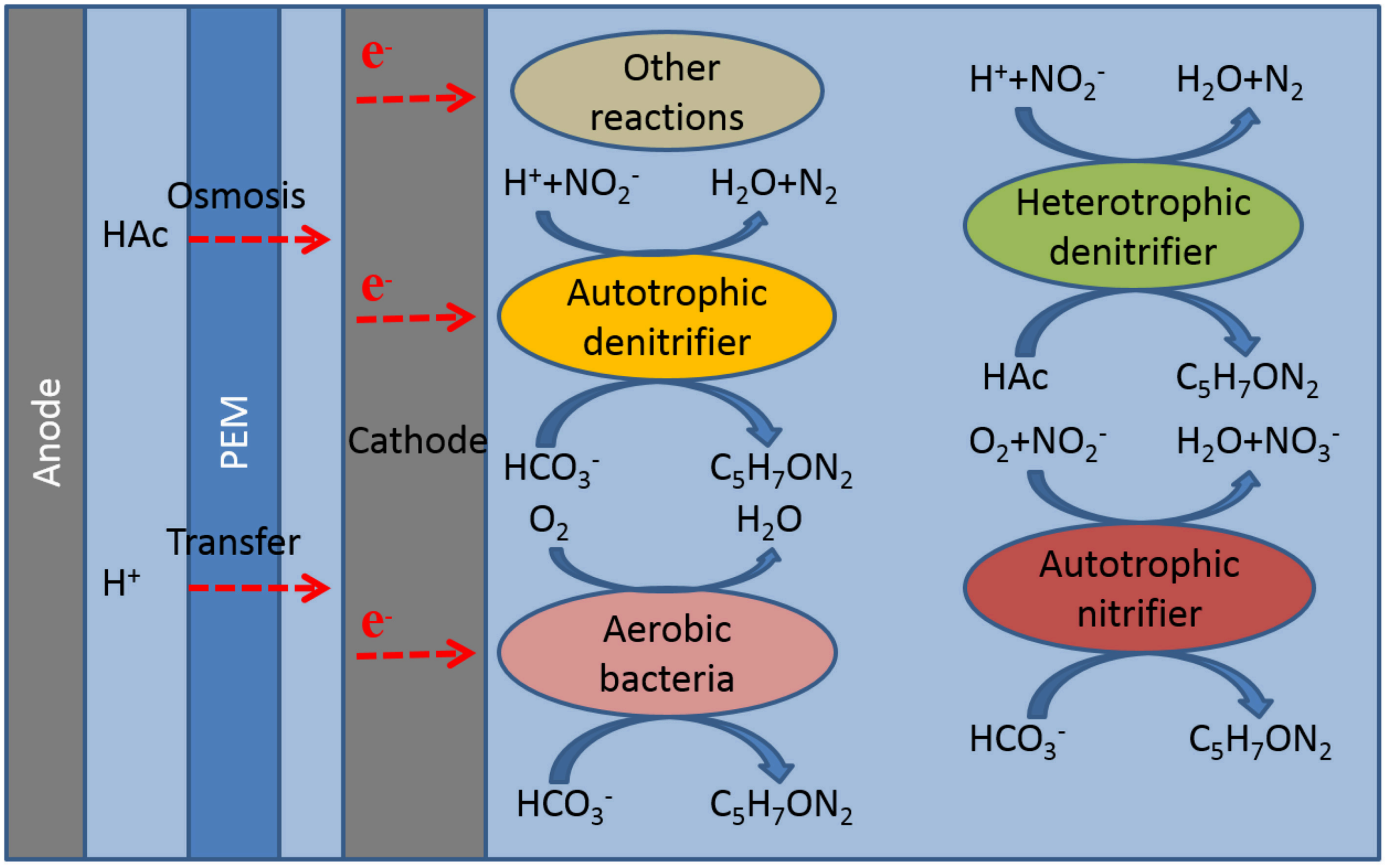
**Mechanism of the cathode chamber**.

From the analysis of the microbial community composition and the experimental results, we speculated for possible reactions in the cathode of MFC (Figure 4).

1. Autotrophic denitrification: Known as autotrophic electrotrophs with an electrode as the electron donor in the cathode of MFC (Virdis et al., 2008; Puig et al., 2011). *Afipia* and *Thiobacillus* directly contributed to autotrophic denitrification. Several researchers also demonstrated the autotrophic bacterium dominated in the cathode microbial community (Wrighton et al., 2010).
2. Heterotrophic denitrification: Organic matter was not added in the cathode, however, about 25 mg L^−1^ of COD was detected, so the existence of heterotrophic denitrifying bacteria *Ignavibacterium* might be caused by sodium acetate in the anode permeate through proton membrane to the cathode (Kim et al., 2007; Chae et al., 2008; Okamoto et al., 2013). Xiao et al. indicated the heterotrophic bacterium survival in the autotrophic denitrifying cathode of MFC (Xiao et al., 2015).
3. Autotrophic nitrification: The high convert of nitrite to nitrate in this experiment and autotrophic nitrifying bacteria *Devosia* indicated nitrification happened in the cathode (Vanparys et al., 2005).
4. Oxygen reduction: The cathode coulombic efficiency over 100% in most of the experiments and aerobic *Truepera* and *Pelomonas* indicated oxygen as the electron acceptor in the cathode (Albuquerque et al., 2005; Chandra et al., 2011; Xie et al., 2011).
5. Other electrochemical reactions: Although oxygen was not detected in cathode through the whole experiment, the high cathode coulombic efficiency and nitrification rate showed the presence of other oxidant. We speculated that the oxidant might be produced from the other electrochemical reactions.

## Conclusions

Nitrite reduction has been shown to be a bio-catalytic process in denitrifying MFCs that could produce bioelectricity. Nitrite could be oxidized in the cathode via biological or electrochemical processes; the maximum TN removal rate of 54.80 ± 0.01 g m^−3^ d^−1^ was obtained. It would be desirable for the TN removal but not electricity generation at a lower external resistance in MFC. An analysis of bio-cathode biofilms indicated *Proteobacteria* was the dominant species, accounting for 35.72%. *Afipia and Thiobacillus* mainly benefit to autotrophic denitrification in MFC. *Truepera, Devosia, and Pelomonas* might contribute to electricity generation. We speculated for possible reactions in the cathode according to the microbial community analysis and the experimental results.

## Author contributions

HZ and JZ designed the experiment, and supervised conduct of the experiment. HZ supervised the data collection. HZ and JZ drafted the manuscript, FL and XL contributed substantially to the revision.

### Conflict of interest statement

The authors declare that the research was conducted in the absence of any commercial or financial relationships that could be construed as a potential conflict of interest.

## References

[B1] Albuquerque L. Simoes C. Nobre M. F. Pino N. M. Battista J. R. Silva M. T.. (2005). *Truepera* radiovictrix gen. nov., sp. nov., a new radiation resistant species and the proposal of *Trueperaceae fam*. nov. FEMS Microbiol. Lett. 247, 161–169. 10.1016/j.femsle.2005.05.002 15927420

[B2] APHA (1998). Standard Methods for the Examination of Water and Wastewater, 20th Edn. Washington, DC: United Book Press.

[B3] Bond D. R. Lovley D. R. (2003). Electricity production by Geobacter sulfurreducens attached *to* electrodes. Appl. Environ. Microbiol. 69, 1548–1555. 10.1128/AEM.69.3.1548-1555.2003 12620842 PMC150094

[B4] Cha J. Choi S. Yu H. N. Kim H. Kim C. (2010). Directly applicable microbial fuel cells in aeration tank for wastewater treatment. Bioelectrochemistry 78, 72–79. 10.1016/j.bioelechem.2009.07.009 19674944

[B5] Chae K. J. Choi M. Ajayi F. F. Park W. Chang I. S. Kim I. S. (2008). Mass transport through a proton exchange membrane (Nafion) in microbial fuel cells. Energy Fuels 22, 169–176. 10.1021/ef700308u

[B6] Chandra R. Bharagava R. N. Kapley A. Purohit H. J. (2011). Bacterial diversity, organic pollutants and their metabolites in two aeration lagoons of common effluent treatment plant (CETP) during the degradation and detoxification of tannery wastewater. Bioresour. Technol. 102, 2333–2341. 10.1016/j.biortech.2010.10.087 21075615

[B7] Clauwaert P. Rabaey K. Aelterman P. de Schamphelaire L. Pham T. H. Boeckx P.. (2007). Biological denitrification in microbial fuel cells. Environ. Sci. Technol. 41, 3354–3360. 10.1021/es062580r 17539549

[B8] Deng Y. Zhang X. Miao Y. Hu B. (2016). Exploration of rapid start-up of the CANON process from activated sludge inoculum in a sequencing biofilm batch reactor (SBBR). Water Sci. Technol. 73, 535–542. 10.2166/wst.2015.518 26877035

[B9] Desloover J. Puig S. Virdis B. Clauwaert P. Boeckx P. Verstraete W.. (2011). Biocathodic nitrous oxide removal in bioelectrochemical systems. Environ. Sci. Technol. 45, 10557–10566. 10.1021/es202047x 22070656

[B10] Guisasola A. Jubany I. Baeza J. A. Carrera J. Lafuente L. (2005). Respirometric estimation of the oxygen affinity constants for biological ammonium and nitrite oxidation. J. Chem. Technol. Biotechnol. 80, 388–396. 10.1002/jctb.1202

[B11] Karanasios K. A. Vasiliadou I. A. Pavlou S. Vayenas D. V. (2010). Hydrogenotrophic denitrification of potable water: a review. J. Hazard. Mater. 180, 20–37. 10.1016/j.jhazmat.2010.04.090 20471745

[B12] Katuri K. P. Scott K. Head I. M. Picioreanu C. Curtis T. P. (2011). Microbial fuel cells meet with external resistance. Bioresour. Technol. 102, 2758–2766. 10.1016/j.biortech.2010.10.147 21146983

[B13] Kelly D. P. Wood A. P. (2000). Reclassification of some species of *Thiobacillus* to the newly designated genera Acidithiobacillus gen. nov., Halothiobacillusgen. nov. and Thermithiobacillus gen. nov. Int. J. Syst. Evol. Microbiol. 50, 511–516. 10.1099/00207713-50-2-511 10758854

[B14] Kim J. R. Cheng S. Oh S. E. Logan B. E. (2007). Power generation using different cation, anion and ultrafiltration membranes in microbial fuel cells. Environ. Sci. Technol. 41, 1004–1009. 10.1021/es062202m 17328216

[B15] Kondaveeti S. Lee S. H. Park H. D. Min B. (2014). Bacterial communities in a bioelectrochemical denitrification system: the effects of supplemental electron acceptors. Water Res. 51, 25–36. 10.1016/j.watres.2013.12.023 24388828

[B16] La Scola B. Mallet M. N. Grimont P. A. Raoult D. (2002). Description of *Afipia birgiae* sp. nov. and *Afipia massiliensis* sp. nov. and recognition of *Afipia felis* genospecies A. Int. J. Syst. Evol. Microbiol. 52, 1773–1782. 10.1099/00207713-52-5-1773 12361286

[B17] Li J. T. Zhang S. H. Hua Y. M. (2013). Performance of denitrifying microbial fuel cell subjected to variation in pH, COD concentration and external resistance. Water Sci. Technol. 68, 251–256. 10.2166/wst.2013.250 23823562

[B18] Li W. Q. Zhang S. H. Chen G. Hua Y. M. (2014). Simultaneous electricity generation and pollutant removal in microbial fuel cell with denitrifying biocathode over nitrite. Appl. Energy 126, 136–141. 10.1016/j.apenergy.2014.04.015

[B19] Logan B. E. Aelterman P. Hamelers B. Rozendal R. Schröder U. Keller J.. (2006). Microbial fuel cells: methodology and technology. Environ. Sci. Technol. 40, 5181–5192. 10.1021/es0605016 16999087

[B20] Okamoto H. Kawamura K. Nishiyama T. Fujii T. Furukawa K. (2013). Development of a fixed-bed anammox reactor with high treatment potential. Biodegradation 24, 99–110. 10.1007/s10532-012-9561-x 22684212 PMC3553412

[B21] Puig S. Marc S. Vilar-Sanz A. Cabré M. Bañeras L. L. Colprim J.. (2011). Autotrophic nitrite removal in the cathode of microbial fuel cells. Bioresour. Technol. 102, 4462–4467. 10.1016/j.biortech.2010.12.100 21262566

[B22] Van Doan T. Lee T. K. Shukla S. K. Tiedje J. M. Park J. (2013). Increased nitrous oxide accumulation by bioelectrochemical denitrification under autotrophic conditions: kinetics and expression of denitrification pathway genes. Water Res. 47, 7087–7097. 10.1016/j.watres.2013.08.041 24210359

[B23] Vanparys B. Heylen K. Lebbe L. Vos P. D. (2005). *Devosia limi* sp. nov., isolated from a nitrifying inoculums. Int. J. Syst. Evol. Microbiol. 55, 1997–2000. 10.1099/ijs.0.63714-0 16166701

[B24] Virdis B. Rabaey K. Rozendal R. A. Yuan Z. Keller J. (2010). Simultaneous nitrification, denitrification and carbon removal in microbial fuel cells. Water Res. 44, 2970–2980. 10.1016/j.watres.2010.02.022 20303136

[B25] Virdis B. Rabaey K. Yuan Z. Keller J. (2008). Microbial fuel cells for simultaneous carbon and nitrogen removal. Water Res. 42, 3013–3024. 10.1016/j.watres.2008.03.017 18466949

[B26] Wang Q. Feng C. Zhao Y. Hao C. (2009). Denitrification of nitrate contaminated groundwater with a fiber-based biofilm reactor. Bioresour. Technol. 100, 2223–2227. 10.1016/j.biortech.2008.07.057 19013791

[B27] Wrage N. Velthof G. L. Van Beusichem M. L. Oenema O. (2001). Role of nitrifier denitrification in the production of nitrous oxide. Soil Biol. Biochem. 33, 1723–1732. 10.1016/S0038-0717(01)00096-7

[B28] Wrighton K. C. Virdis B. Clauwaert P. Read S. T. Daly R. A. Boon N.. (2010). Bacterial community structure corresponds to performance during cathodic nitrate reduction. ISME J. 4, 1443–1455. 10.1038/ismej.2010.66 20520654

[B29] Xiao Y. Zheng Y. Wu S. Yang Z. H. Zhao F. (2015). Bacterial community structure of autotrophic denitrification biocathode by 454 pyrosequencing of the 16S rRNA Gene. Environ. Microbiol. 69, 492–499. 10.1007/s00248-014-0492-4 25213655

[B30] Xie S. Liang P. Chen Y. Xia X. Huang X. (2011). Simultaneous carbon and nitrogen removal using an oxic/anoxic-biocathode microbial fuel cells coupled system. Bioresour. Technol. 102, 348–354. 10.1016/j.biortech.2010.07.046 20685109

[B31] Yamada T. Sekiguchi Y. S. Imachi H. Ohashi A. Harada H. Kamagata Y. (2006). Anaerolinea thermolimosa sp. nov., *Levilinea saccharolytica* gen. nov., sp. nov. and *Leptolinea tardivitalis* gen. nov., sp. nov., novel filamentous anaerobes, and description of the new classes *Anaerolineae classis* nov. and *Caldilineae classis* nov. in the bacterial phylum Chloroflex. Int. J. Syst. Evol. Microbiol. 56, 1331–1340. 10.1099/ijs.0.64169-0 16738111

[B32] Zhang F. He Z. (2012). Simultaneous nitrification and denitrification with electricity generation in dual-cathode microbial fuel cells. Chem. Technol. Biotechnol. 87, 153–159. 10.1002/jctb.2700

[B33] Zhang L. Zhu X. Li J. Liao Q. Ye D. D. (2011). Biofilm formation and electricity generation of a microbial fuel cell started up under different external resistances. Power Sources 196, 6029–6035. 10.1016/j.jpowsour.2011.04.013

[B34] Zhao Y. Feng C. Wang Q. Yang Y. Zhang Z. Sugiura N. (2011). Nitrate removal from groundwater by cooperating heterotrophic with autotrophic denitrification in a biofilm-electrode reactor. J. Hazard. Mater. 192, 1033–1039. 10.1016/j.jhazmat.2011.06.008 21724327

